# Understanding the contribution of neural and physiological signal variation to the low repeatability of emotion-induced BOLD responses^☆^

**DOI:** 10.1016/j.neuroimage.2013.10.015

**Published:** 2014-02-01

**Authors:** I. Lipp, K. Murphy, R.G. Wise, X. Caseras

**Affiliations:** a Cardiff University Brain Research Imaging Centre (CUBRIC), School of Psychology, Cardiff University, UK; b MRC Centre for Neuropsychiatric Genetics and Genomics, Institute of Psychological Medicine and Clinical Neurosciences, Cardiff University, UK

**Keywords:** fMRI, BOLD, Repeatability, Emotion processing, Physiological noise correction

## Abstract

Previous studies have reported low repeatability of BOLD activation measures during emotion processing tasks. It is not clear, however, whether low repeatability is a result of changes in the underlying neural signal over time, or due to insufficient reliability of the acquired BOLD signal caused by noise contamination. The aim of this study was to investigate the influence of “cleaning” the BOLD signal, by correcting for physiological noise and for differences in BOLD responsiveness, on measures of repeatability.

Fifteen healthy volunteers were scanned on two different occasions, performing an emotion provocation task with faces (neutral, 50% fearful, 100% fearful) followed by a breath-hold paradigm to provide a marker of BOLD responsiveness. Repeatability of signal distribution (spatial repeatability) and repeatability of signal amplitude within two regions of interest (amygdala and fusiform gyrus) were estimated by calculating the intraclass correlation coefficient (ICC).

Significant repeatability of signal amplitude was only found within the right amygdala during the perception of 50% fearful faces, but disappeared when physiological noise correction was performed. Spatial repeatability was higher within the fusiform gyrus than within the amygdala, and better at the group level than at the participant level. Neither physiological noise correction, nor consideration of BOLD responsiveness, assessed through the breath-holding, increased repeatability.

The findings lead to the conclusion that low repeatability of BOLD response amplitude to emotional faces is more likely to be explained by the lack of stability in the underlying neural signal than by physiological noise contamination. Furthermore, reported repeatability might be a result of repeatability of task-correlated physiological variation rather than neural activity. This means that the emotion paradigm used in this study might not be useful for studies that require the BOLD response to be a stable measure of emotional processing, for example in the context of biomarkers.

## Highlights

• We investigated repeatability of BOLD responses during a widely used emotion task.
• Repeatability of signal amplitude was low in the amygdala and in the fusiform gyrus.
• Spatial repeatability was higher within the fusiform gyrus than within the amygdala.
• Physiological noise correction did not improve any of the repeatability measures.
• The BOLD response to this emotion task might not be useful as a biomarker.

## Introduction

Functional magnetic resonance imaging (fMRI) is a widely used tool for studying emotion in the human brain. Recent research in this area has highlighted the crucial role of the amygdala in the experience of emotion (Costafreda et al., 2008, Phan et al., 2002). Activity in the amygdala, measured through BOLD fMRI, has been suggested as a biomarker for different psychiatric disorders (Phillips et al., 2003). The assumption underlying the proposed biomarker is that the signal change measured in the amygdala is sufficiently strongly driven by inter-individual differences in neural activity induced by experimental challenges compared to other sources of fluctuations, or noise. However, previous studies have reported low repeatability of amygdala BOLD responses during emotion provocation (e.g. Johnstone et al., 2005, Manuck et al., 2007, Plichta et al., 2012), arguing against the suitability of this measure as a biomarker.

In order to obtain a quantitative estimate of repeatability, the intraclass correlation coefficient (ICC) is used most commonly (Caceres et al., 2009, Shrout and Fleiss, 1979), which reflects the ratio between the data variance of interest (inter-subject BOLD differences) and the total data variance. One way to use the ICC in the context of brain imaging is to extract the mean percent signal change from repeated measures in an area of interest and calculate the ICC for the obtained values, estimating repeatability of the signal amplitude (e.g. Johnstone et al., 2005). Another approach is to obtain spatial ICCs for particular regions of interest (ROIs). In this case, each voxel within the ROI is considered during ICC calculation, and for each participant, a single ICC is obtained. These ICCs reflect the repeatability of the signal's spatial distribution (Plichta et al., 2012). Repeatability of the activity within the amygdala has been investigated in both ways, resulting in low to medium ICC values for signal amplitude (Johnstone et al., 2005, Manuck et al., 2007) and low values for signal distribution (Plichta et al., 2012, Stark et al., 2004, Van den Bulk et al., 2013). Spatial repeatability at the group-level has been shown to be higher than that at the individual level (Plichta et al., 2012).

Low repeatability of BOLD responses can result from two factors or their combination: a) brain responses are too unstable to show temporal reliability (neural response variability); b) brain responses remain stable but cannot be measured accurately (measurement variability). If the former is true, BOLD responses in the amygdala would not be suitable as a stable biomarker for psychiatric disorders. However, if low repeatability is due to measurement error, or noise, there is the potential for improvement by refining the data acquisition and analysis methods.

One important factor that might introduce noise in the measurement of BOLD within the amygdala is the physiological reaction that also accompanies emotional responses. The recorded BOLD signal time-course is influenced by changes in breathing and heart rate (Birn et al., 2006, Birn et al., 2009, Chang and Glover, 2009, Harvey et al., 2008, Wise et al., 2004). By recording and accounting for these changes, it is suggested that a “cleaner” measure of neural activation can be acquired (Chang and Glover, 2009). Another important aspect is that the stimulus-induced BOLD contrast depends on the hemodynamic responsiveness, blood volume and T2* (Clare et al., 2001), itself dependent on the local field gradients (Murphy et al., 2011, Thomason et al., 2005). Day-to-day differences in these factors might lead to day-to-day differences in the measured BOLD response even if the underlying neural activity were the same. One method that has been introduced to measure BOLD signal responsiveness independent of task is to increase the arterial CO_2_ level with a hypercapnic challenge (artificially regulating the CO_2_ level of the environment) or with voluntary breath-holding. Using this method, cerebral blood flow (CBF) is transiently increased without affecting the oxygen consumption rate throughout the whole brain (Thomason et al., 2005), producing a concomitant increase in BOLD signal.

The aim of this study was to investigate the between-session repeatability of the BOLD measure during the perception of emotional faces, and to assess whether repeatability can be enhanced by applying physiological noise correction and measures of BOLD (vascular) responsiveness. Following previous studies, the amygdala was chosen as the main region of interest. The fusiform gyrus was selected as an additional region of interest because it has been reported to be involved in the perception of faces in general (e.g. McCarthy et al., 1997) as well as in emotion processing (e.g. Vuilleumier and Pourtois, 2007), and was expected to provide strong BOLD signal responses to our experimental stimuli.

## Methods

### Participants

Fifteen (8 male) participants with a mean age of 24 (*SD* = 1.6) voluntarily took part in the study having given informed, written consent. They undertook the scanning protocol twice with a mean interval of 23 days (range: 15–34; *SD* = 4.8). One participant was excluded from all analysis involving BOLD responsiveness due to a CO_2_ trace that was unusable for analysis. The study was approved by the Cardiff University School of Medicine Research Ethics Committee.

### Tasks

#### Emotion provocation task

The emotion perception paradigm was adapted from Surguladze et al. (2010) and has been widely used in clinical research to investigate emotional reactivity in mental disorders. Emotional faces selected from the Ekman and Friesen pool (FEEST, Young et al., 2002) of 10 identities (5 male: EM, JJ, NR, PE, WF; 5 female: C, MF, MO, PF, SW) were presented, showing 50% morphed fear–neutral expression, 100% fearful expressions or 25% morphed happy–neutral as neutral expressions (Morris et al., 1998). Each identity was presented six times, and each identity–emotion combination was presented twice during the task. In order to maintain participant attention on the presented stimuli, they were instructed to perform a male/female categorization task responding with a button press (index finger press for male, middle finger for female). Stimuli were presented for 2 s with a variable pseudorandomized inter-stimulus interval (fixation cross) between 3 and 8 s. Stimulus order was pseudorandomized with the same intensity of emotion not occurring more than twice in a row. The task took 8 min to complete.

#### Breath-holding task

The breath-holding task was adapted from Murphy et al. (2011). During the task, breathing instructions were presented on the screen, guiding the participant through six cycles of breath-holding and recovery, each with four different phases: paced breathing (alternating breathing in and breathing out for 3 s each) for 18 s, end-expiration breath-holding for 15 s, exhalation, and final recovery (spontaneous breathing with no breathing instructions) for 15 s. The task took 5 min to complete.

### Recordings

The participants underwent gradient-echo echo-planar imaging at 3 T (GE HDx MRI System) with a T2* weighted imaging sequence (TR = 3 s, TE = 35 ms, matrix = 64 × 64, FOV/slice = 220 mm, flip = 90, 53 slices of 2 mm with a 1 mm slice gap acquired in an interleaved order) using an eight-channel receive-only head coil. The orientation of the axial slices was parallel to the AC–PC line. During the emotion provocation task 154 functional image volumes were obtained, and 108 volumes were acquired during the breath-holding task. The tasks were presented using Presentation (Neurobehavioral Systems, Albany, CA) and rear-projected onto a screen behind the participant's head that was visible through a mirror mounted on the head RF coil.

A T_1_ weighted whole-brain structural scan was also acquired for purposes of image registration (1 × 1 × 1 mm resolution, 256 × 256 × 176 matrix size). The structural image was only acquired during session 1, and this image was used for registration for the functional images of session 1 and session 2.

During both scanning sessions, physiological parameters were recorded: a) the cardiac cycle was recorded using a pulse-oximeter placed on the left index finger, b) a respiration trace was recorded with a pneumatic belt around the chest, c) end-tidal carbon dioxide (Pet_CO2_) and end-tidal oxygen (Pet_O2_) were recorded using a nasal cannula attached to rapidly responding gas analyzers (AEI Technologies, PA) to provide representative measures of arterial partial pressures of both gases.

### Data preprocessing and analysis

BOLD responses during the emotion paradigm were analyzed with and without physiological noise correction of the BOLD fMRI time-series data. This correction consisted of: first applying correction of cardiac and respiratory artifacts (RETROICOR, Glover et al., 2000) using two cardiac, two respiratory and one interaction component, followed by regressing out the variance related to carbon dioxide (Pet_CO2_) level, oxygen (Pet_O2_) level, heart rate (HR) and respiratory volume per time (RVT; Birn et al., 2006) using a general linear model framework. Both steps were performed using Matlab (The MathWorks Inc., vs. R2011a). Physiological noise correction was performed prior to preprocessing.

Both datasets (uncorrected and physiological noise corrected) were subsequently analyzed using FEAT (FMRIB Expert Analysis Tool, v5.98, http://www.fmrib.ox.ac.uk/fsl, Oxford University, UK). Preprocessing steps before model fitting were applied to each participant's time-series, and included: highpass filtering of the data (100 s temporal cutoff), non-brain removal using BET (Smith, 2002), “MCFLIRT” motion correction (Jenkinson et al., 2002), spatial smoothing with a Gaussian kernel of full-width–half-maximum 5 mm and fieldmap-based EPI unwarping using PRELUDE + FUGUE (Jenkinson, 2003, Jenkinson, 2004; for one person this was not performed due to problems during the acquisition of the fieldmaps). Due to the long TR (3 s), slice-time correction was performed as recommended by Sladky et al. (2011). Functional images were registered using FLIRT (Jenkinson and Smith, 2001) in a first step to the structural image with 6 degrees of freedom, and in the second step to the Montreal Neurological Institute (MNI) space with 12 degrees of freedom and FNIRT non-linear (10 mm) warp (Andersson et al., 2007a, Andersson et al., 2007b).

To model the emotion provocation task, three event types were defined, one for each emotion condition (i.e. neutral, 50% fear and 100% fear expressions); the fixation cross periods were used as the baseline. The model was convolved with the hemodynamic response function (gamma convolution), and the same temporal filtering was applied to the model as to the data. Temporal derivatives of the event-type regressors were included as regressors of no interest. The main effects for neutral, 50% fear and 100% fear were evaluated, as well as the contrast fear (average of 50 and 100% fear) > neutral. Group average maps were created with a fixed effects model using FLAME. The Z (Gaussianised T/F) statistic images were thresholded using clusters determined by Z > 2.3 and a (corrected) cluster significance threshold of *p* < .05 (Worsley, 2001).

A region of interest (ROI) analysis was performed for the amygdala and fusiform gyrus for both hemispheres. Anatomical masks were taken from the WFU-PickAtlas (Version 3.0.4, Wake Forest University, School of Medicine, Winston-Salem, North Carolina, www.ansir.wfubmc.edu). Percent signal changes for whole brain maps and for ROIs were computed. For each region of interest, a repeated measures ANOVA was calculated for percent signal change within the ROI as the dependent variable, with the emotion condition and hemisphere as independent variables. Calculations were performed using SPSS (IBM SPSS v.19), with a chosen significance level of *p* < .05.

The breath-holding task was used to obtain a BOLD responsiveness map for each participant, based on BOLD signal changes. The recorded end-tidal CO_2_ trace obtained during the breath-holding task was demeaned and entered as a regressor in a GLM along with its temporal derivative. In order to obtain a measure of BOLD signal change per unit change of CO_2_, the range of the fitted time series (2nd–98th percentile to minimize the influence of outliers) was divided by the range of the end-tidal CO_2_ trace (2nd–98th percentile). The resulting maps were concatenated across participants before temporal demeaning and then entered as a voxelwise regressor in the group-level analysis (Murphy et al., 2011). Spatial repeatability analyses at the group level for the emotion provocation task were then conducted on the group activation maps with and without the inclusion of the BOLD responsiveness as a covariate across the group.

### Repeatability analysis

**Fig. 1:**
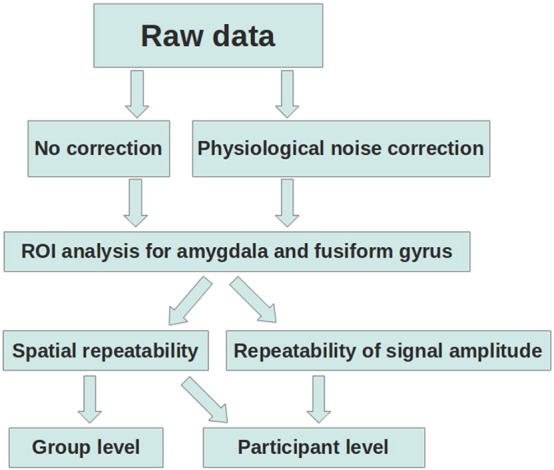
Overview of repeatability analysis. Raw data was either corrected for physiological noise or left uncorrected. For each ROI, spatial repeatability (at the group and at the participant level) as well as repeatability of the signal amplitude (at the participant level) was estimated.

The intraclass correlation coefficient (ICC(3,1); Shrout and Fleiss, 1979) was used as a measure of repeatability. The ICC variant ICC(3,1) was chosen because it removes mean difference over the two times of measurement. For an overview of the repeatability analyses see Fig. 1. ICCs were interpreted according to commonly used guidelines (Cicchetti, 2001; see also e.g. Van den Bulk et al., 2013) that classify values of < .41 as poor, values between .41 and .59 as fair, values between .60 and .74 as good and values > .74 as excellent.

#### Repeatability of the activation at the participant level: Repeatability of signal amplitude between sessions

The mean percent BOLD signal change for each ROI was extracted and ICCs as well as descriptive statistics were calculated using Matlab. ICCs were tested with a significance level set at *p* < .05. An additional analysis was conducted in order to control for changes in BOLD responsiveness that might influence the percent signal change in the emotion task. For each region, a linear regression was performed between percent signal change obtained during the breath-hold task and percent signal change obtained during the emotion task.

The residuals from this regression constitute the percent signal change during the emotion task that cannot be explained by BOLD responsiveness. These values were then taken to compute ICCs having accounted for BOLD responsiveness. Since day-to-day variation in signal dropout within the ROIs could potentially influence the obtained repeatability measures, ICCs were also calculated for the mean signal intensity over time and space within the ROIs. Mean signal intensity was obtained from the time series image after all preprocessing steps have been performed.

#### Repeatability of the activation at the participant level: Spatial repeatability between sessions

For each participant and each region of interest, as well as for the whole brain, a spatial ICC was calculated for the unthresholded Z-score maps within the respective region from the two sessions.

#### Repeatability of the activation at the group level: Spatial repeatability

Spatial repeatability at the group-level was estimated by using the group level unthresholded Z-score maps for session 1 and session 2 and computing a spatial ICC over all voxels within each of the regions of interest.

#### Task-related variance in physiological parameters

We investigated the relationship between the stimulus paradigm and the recorded physiological parameters to uncover potential effects of the task on the volunteer's physiology which may in turn influence BOLD signal responses. For each participant, a linear regression was performed between the HRF convolved stimulus time series and the physiological signals. These included the CO_2_, O_2_, HR, and RVT convolved with a HRF as well as the RETROICOR regressors. Since the latter differ significantly for each acquired slice, the regressor was generated for a slice passing through the amygdala (this was defined for each participant separately by visual inspection). The amount of task-correlated variation in physiological measures was defined as the shared variance (R^2^) between the physiological regressors and the expected hemodynamic stimulus response model for each stimulus condition separately as well as for the complete task (HRF-convolved stimulus time series were summed with equal weight) and for the contrast fear > neutral (time series for the both fear conditions were summed and the time series for neutral stimuli was subtracted).

## Results

### Behavioral results

**Fig. 2:**
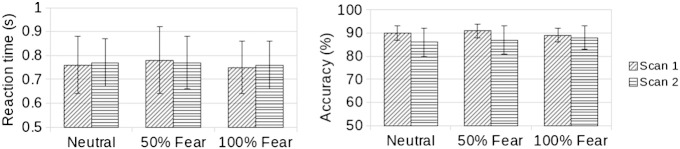
Behavioral results. Mean and standard deviation (error bar) of reaction time (left), and accuracy (right) are shown for each scan and condition.

All participants responded to more than 97% (and over 88% correctly) of the face stimuli during both scanning sessions, indicating that they paid attention to the task. There were no differences in mean reaction times (F[1,28] = 0.11, *ns*) between scanning sessions but there was a trend for higher accuracy in the first scanning session than in the second session (F[1,28] = 4.61, *p* = .05). Fear intensity did not have an influence on the measures of task performance (accuracy: F[2,28] = 1.01, *ns*; reaction time: F[2,28] = 1.45, *ns*; see Fig. 2).

### Task-related activation

#### ROI analysis

**Fig. 3:**
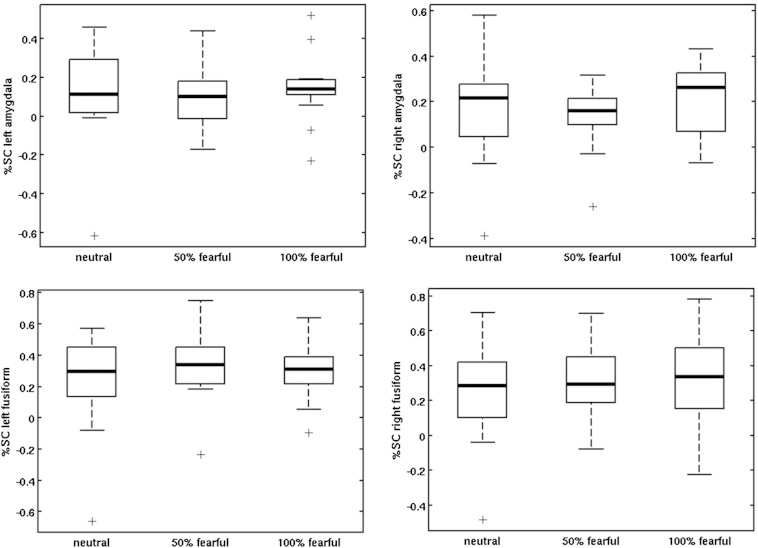
ROI activation. Percent signal change within the left amygdala (top left), right amygdala (top right), left fusiform gyrus (bottom left) and right fusiform gyrus (bottom right) is shown for the three fear intensity conditions in session 1.

Percent signal changes relative to baseline were extracted for the amygdala and fusiform gyrus. In both ROIs, a large variance between participants was observed. As expected, mean signal changes were higher within the fusiform gyrus than within the amygdala. For descriptive statistics and between-session differences see Table 1 and Fig. 3. Results for ANOVAS (influence of condition and hemisphere on percent signal change in the ROIs) can be found in the Supplementary material (Task-related activation – ROI analysis).

**Table 1:** ROI analysis for uncorrected data. For each of the ROIs, mean and standard deviation of the percent signal change for both scanning sessions (scan 1 and scan 2), the significance of the between-session difference (paired t-test), the repeatability of the value (ICC) and its significance are provided.

	No correction				Physiological noise correction			
Area/condition	*M* (*SD*) scan 1	*M* (*SD*) scan 2	*p* (diff)	ICC (*p*)	*M* (*SD*) scan 1	*M* (*SD*) scan 2	*p* (diff)	ICC (*p*)
*Left amygdala*								
Neutral	0.08 (0.25)	0.13 (0.20)	.46	.12 (.33)	0.07 (0.16)	0.13 (0.22)	.42	.01 (.49)
50% fearful	0.06 (0.17)	0.02 (0.30)	.61	.05 (.43)	0.10 (0.20)	0.06 (0.23)	.65	− .04
100% fearful	0.14 (0.20)	0.14 (0.16)	.99	.37 (.08)	0.09 (0.20)	0.12 (0.18)	.68	.29 (.14)
Fear > Neutral	0.04 (0.20)	− 0.04 (0.19)	.37	− .56	0.04 (0.15)	− 0.03 (0.24)	.52	− .68

*Right amygdala*								
Neutral	0.17 (0.23)	0.10 (0.22)	.38	.11 (.35)	0.14 (0.16)	0.09 (0.22)	.51	.06 (.41)
50% fearful	0.14 (0.16)	0.04 (0.26)	.10	.48 (.03)	0.16 (0.18)	0.06 (0.18)	.12	.25 (.17)
100% fearful	0.21 (0.17)	0.07 (0.15)	.01	.33 (.11)	0.14 (0.17)	0.05 (0.13)	.07	.19 (.24)
Fear > Neutral	0.03 (0.17)	− 0.03 (0.18)	.42	− .51	0.03 (0.09)	− 0.04 (0.17)	.33	− .42

*Left fusiform*								
Neutral	0.21 (0.32)	0.25 (0.25)	.74	.12 (.32)	0.17 (0.24)	0.15 (0.20)	.83	.04 (.45)
50% fearful	0.31 (0.22)	0.13 (0.35)	.15	− .28	0.25 (0.28)	0.11 (0.20)	.17	− .21
100% fearful	0.30 (0.18)	0.20 (0.22)	.28	− .38	0.20 (0.20)	0.12 (0.17)	.32	− .24
Fear > neutral	0.13 (0.32)	− 0.06 (0.20)	.09	− .13	0.08 (0.18)	− 0.02 (0.12)	.10	− .05

*Right fusiform*								
Neutral	0.29 (0.30)	0.31 (0.24)	.82	.09 (.37)	0.26 (0.23)	0.19 (0.24)	.41	.12 (.32)
50% fearful	0.33 (0.20)	0.16 (0.30)	.11	− .06	0.30 (0.25)	0.12 (0.21)	.05	− .03
100% fearful	0.34 (0.25)	0.27 (0.20)	.44	− .14	0.25 (0.26)	0.17 (0.16)	.33	− .10
Fear > neutral	0.08 (0.27)	− 0.07 (0.25)	.14	− .04	0.05 (0.12)	− 0.03 (0.17)	.13	.11 (.35)

#### Exploratory whole-brain analysis

Widespread significant BOLD activation was found during the presentation of the facial stimuli as compared to the baseline (for activation maps see Supplementary Figs. S1–S3). Using the whole brain approach, no areas appeared to be more strongly activated during the presentation of fearful faces (both intensities combined) than the presentation of neutral faces in either of the two scanning sessions and independent of physiological noise correction.

### Repeatability of the activation at the participant level

#### Repeatability of signal amplitude

In order to obtain a value for repeatability for each ROI, percent signal changes for the amygdala and fusiform gyrus in both hemispheres were extracted, and ICCs were computed. The highest repeatability was found in the right amygdala for moderately fearful faces with an ICC of .48 (*p* = .03). The same analysis was performed using the dataset corrected for physiological noise for which no significant ICC was found (for all values see Table 1). Regressing out breath-hold based BOLD responsiveness measures did not increase the ICCs (see Tables S1 and S2 in the Supplementary material).

In order to investigate whether signal dropout was the cause of this low repeatability in the regions of interest, the ICC was also calculated for the mean signal intensity over the whole length of the scan in these areas (see the Methods section). The ICCs for the left and the right amygdala were .82 (*p* < .001) and .92 (*p* < .001), respectively; and .89 (*p* < .001) for the left fusiform, and .76 (*p* = < .001) for the right fusiform, indicating excellent repeatability of mean BOLD signal intensity.

#### Spatial repeatability

In order to investigate whether the distribution rather than overall amplitude of BOLD activation is repeatable, a spatial ICC was calculated for each person, using the two Z maps within each region of interest. Table 2 shows the mean and standard deviation of the obtained ICCs. The ICCs for the fusiform indicate fair spatial repeatability for the main effect fear. Spatial repeatability in the amygdalae was poor for the contrast fear for the uncorrected dataset (ICC > 20) but could be slightly improved for the physiological noise corrected dataset (ICC > .40).

**Table 2:** 1st level spatial repeatability. Voxel-based ICCs for participant level SC-maps of the main effect fear, and the contrast fear > neutral. ICCs were converted to z(r) using the Fisher-z transformation before averaging across participants.

		Left amygdala		Right amygdala		Left fusiform		Right fusiform	
Correction		Fear	Fear > neutral	Fear	Fear > neutral	Fear	Fear > neutral	Fear	Fear > neutral
Uncorrected	Mean	.15	.47	.13	.46	.56	.05	.57	.05
	Std.	.34	.08	.28	.08	.19	.20	.27	.20
Physiological noise corrected	Mean	.47	.20	.45	.24	.54	.04	.56	.04
	Std.	.05	.10	.04	.10	.25	.21	.26	.15

### Repeatability of the activation at the group level

For determining the repeatability at the group level, ICCs between the two Z-maps of session 1 and session 2 were calculated for the two regions of interest. For both, the uncorrected and the physiological noise corrected datasets, the ICCs for the amygdala were, in general, poor. Repeatability values for the fusiform gyrus were excellent for both datasets (see Table 3).

**Table 3:** 2nd level repeatability. Voxelbased ICCs for group level Z-maps of the main effect fear, and the contrast fear > neutral.

	Left amygdala		Right amygdala		Left fusiform		Right fusiform	
Correction	Fear	Fear > neutral	Fear	Fear > neutral	Fear	Fear > neutral	Fear	Fear > neutral
Uncorrected without BOLD responsiveness regressor	.18⁎	.43⁎	.39⁎	− .04	.84⁎	.23⁎	.87⁎	.05
Uncorrected with BOLD responsiveness regressor	.17⁎	.40⁎	.32⁎	.01	.84⁎	.18⁎	.86⁎	− .16
Physiological noise corrected without BOLD responsiveness regressor	.34⁎	− .02	.47⁎	− .15	.86⁎	.22⁎	.86⁎	.03
Physiological noise corrected with BOLD responsiveness regressor	.20⁎	.19⁎	.50⁎	− .09	.87⁎	.17⁎	.84⁎	− .03

⁎ *p* < .001.

### Correlation between task and physiology

The shared variance between task-related signal changes and task-related physiological changes was estimated. All task conditions combined could explain on average 8% of the variance, while the three conditions separately could explain on average 12% (neutral stimuli), 9% (50% fear stimuli), 9% (100% fear stimuli). All results for the analysis on task-related variance in physiological parameters can be found in the Supplementary material (Table S3).

## Discussion

The aim of this study was to estimate the repeatability between sessions of BOLD signal changes obtained during a widely used emotion provocation task, and to investigate the effects of physiological noise correction and intra-individual and inter-individual differences in BOLD responsiveness on the repeatability indices. Overall, the repeatability indices of signal amplitude were poor at a participant-level for both the amygdala and the fusiform gyrus. At a group-level, the BOLD signal showed an excellent spatial repeatability within the fusiform gyrus. Correction for physiological noise and accounting for BOLD responsiveness appeared to have little effect on BOLD repeatability for the emotion task.

### BOLD signal repeatability

A widely used emotional paradigm in clinical research (e.g. Almeida et al., 2010, Dannlowski et al., 2009, Rhodes et al., 2007, Surguladze et al., 2010) was implemented here. Participants responded accurately to most of the stimuli presented across emotional conditions. This suggests that overall, participants' level of attention to the facial stimuli during both scanning sessions was adequate. We found high inter-individual variability of BOLD responses within the amygdala and fusiform gyrus. As predicted, the signal was stronger within the fusiform than within the amygdala. A whole-brain analysis revealed widespread activity throughout the brain for the main effect fear, but the frequently reported contrast fearful > neutral faces did not reveal any significant clusters.

Repeatability of signal amplitude was low (< .40) for both regions of interest and effects considered, with an exception of BOLD within the right amygdala during the perception of 50% fearful faces (ICC = .48). Similar ICC values have been reported by Schaefer et al. (2000) in the left amygdala, and by Manuck et al. (2007) in the right amygdala. In our data, however, physiological noise correction decreased this ICC value to .25, which suggests that the detected repeatability might be a result of repeatability of task-correlated physiological variation rather than repeatability of the neural signal in the amygdala. It is also important to notice that even the uncorrected repeatability index was no longer significant when correcting for multiple comparisons.

A cause for these low repeatability indices might lie in inhomogeneous activation within our ROIs. Therefore, the mean signal change within an anatomically defined region might not always be a useful measure for activation, especially when it comes to bigger regions such as the fusiform gyrus. For this reason, we also calculated the spatial repeatability, which provides an indication for how stable the distribution of activation is, independent of the mean signal amplitude. This analysis resulted in fair repeatability measures in the fusiform gyrus bilaterally, and poor results for the amygdala, which is comparable to previously reported outcomes (Plichta et al., 2012, Stark et al., 2004, Van den Bulk et al., 2013). Spatial repeatability could be increased with physiological noise correction to an ICC of about .45, which is still lower than repeatability in the fusiform gyrus. However, it is important to acknowledge that the size of the fusiform gyrus mask favors spatial repeatability compared to the amygdala, since it largely extends beyond the active volume and therefore introduces more variability of the signal within the regarded ROI. Similar results were found when spatial repeatability was calculated for group-level activation. The ICC was higher within the fusiform gyrus (ICCs > .80) than in the amygdala (ICCs < .51), which might also be a result of more variability of group activation level within the fusiform gyrus. Our group-level repeatability in the amygdala is slightly lower than previously reported by Plichta et al. (2012) who used a different emotion task and implemented a block design.

### Influence of physiological noise correction and BOLD responsiveness

Physiological noise correction did not improve repeatability of the BOLD response amplitude. The correction decreased the variance of the fMRI time series in the gray matter by about 15%, which means that physiological parameters are likely to have affected the BOLD response. There are several possible explanations for why the repeatability was not improved. Firstly, if physiological noise did not affect the estimated mean task-related BOLD signal in each session, even by accounting for this physiological noise contribution the BOLD signal changes across the group would not be affected. However, physiological noise correction did lead to an improvement of spatial repeatability for the main effect fear in the amygdala, which suggests that this correction is having a positive effect on the data, albeit small.

Secondly, it is possible that the physiological noise correction did improve the quality of the obtained signal, but also took out variance shared by neural responses and physiological measures (Birn et al., 2009). In fact we showed that physiological factors accounted for approximately 10% of variation in the predicted BOLD signal (i.e. the task regressor), which suggests that the task has an effect on the physiological measures. If this shared variance contributes to repeatability, then physiological noise correction might even decrease the ICC. In fact, this appeared to be the case for the right amygdala.

Thirdly, it is possible that physiological noise correction did improve the quality of the fMRI measure of neural activity, but that this neuronal response is not temporally stable. A lack of stability of the neural response to the stimuli might be partly caused by habituation effects. Significant decrease in activation from session 1 to session 2 was observed in the right amygdala during the perception of fearful images (Table 1). Habituation of the amygdala has been frequently observed, and it seems to be particularly strong in the right hemisphere (Phillips et al., 2001, Wright et al., 2001). The ICC(3,1) that we used to calculate repeatability takes systematic mean differences between the sessions into account, however, individual differences in habituation would still result in low repeatability indices. Additionally, the decrease of signal amplitude leads to a lower signal-to-noise ratio of the BOLD signal, which might affect repeatability. To further investigate whether habituation could have affected our repeatability measure, we re-ran our analyses only considering the first half of the emotional task (for results see Table S4 in the Supplementary material). The rationale behind this approach is that habituation can also happen throughout a scanning session (e.g. Wright et al., 2001) and therefore the first half of either session might be less affected by habituation and may provide a higher signal-to noise ratio (SNR) of the measure. Overall, this approach did not lead to an increase in repeatability, despite producing larger BOLD responses (see Supplementary Table S3). This result also suggests that a lower signal-to-noise ratio is unlikely to be the cause of the low repeatability result.

Adding our breath-hold based measure of BOLD responsiveness to the analysis did not result in higher estimates of repeatability. This suggests that low repeatability is not accounted for by day-to-day differences in our BOLD responsiveness measure.

### Stability of neural response vs. reliability of measure

We showed that performing physiological noise correction and taking BOLD responsiveness into account do not improve repeatability. To investigate the possibility that low repeatability is caused by different amounts of signal dropout in the two sessions, repeatability was also calculated for the whole session-mean signal within the two ROIs. In this case, repeatability indices were high (ICCs > .75). Taking all the results together, the low repeatability does not seem to be caused by physiological noise, signal-dropout or low signal-to-noise ratio of the scanner, but by either other sources of noise or by a lack of temporal stability of the neural response. If the latter is the case, our results suggest caution in considering BOLD responses in the amygdala or fusiform gyrus triggered by emotional expressions as a potential biomarker for psychiatric disorders.

### Limitations and future directions

Participants of this study were all healthy young volunteers and even though a considerable variability in the scores was observed, a more diverse sample (e.g. including patients) might have provided an even broader distribution of activation measures, thus allowing a more sensitive assessment of between session repeatability. Also for this particular sample the contrast fear > neutral did not reveal the oft-reported activation within areas such as the amygdala (Costafreda et al., 2008). It is important to consider that our sample is not comparable to the high anxious samples that are often used in clinical research where this contrast is frequently investigated; although our group should not be much different from any control group included in clinical studies. Also, we implemented a fairly standard task design with regard to stimulus duration, inter-stimulus interval, number of stimuli, and MRI acquisition parameters (e.g. Fu et al., 2007, Lawrence et al., 2004, Surguladze et al., 2005, 2010). As it is the case for most study designs, we did not optimize our acquisition protocol for the detection of amygdala activation but rather for a whole-brain analysis. This might have resulted in reduced power to detect BOLD responses in the amygdala and therefore affected our repeatability measures. However, this triggers the question whether the most common way in which these parameters are used in clinical research represent an appropriate approach.

Another limitation of this study is that we assumed that the physiological noise correction would increase the reliability of the measured neural responses. However, we were able to also demonstrate that physiological variation is correlated with the task, at least in some participants. This means that physiological noise correction may reduce the apparent significance of neurally driven signal changes. Methods need to be developed to distinguish signals of neural origin from non-neuronal physiological effects of the task. However, overall we would recommend performing physiological noise correction since it may increase power to detect task-related activation, as suggested in the current dataset and remains a conservative strategy only being likely to reduce false positives rather than increase them.

Furthermore, it has to be noted that our results apply to a specific emotion provocation task that has been widely used in the mental disorders field. However, a variety of other tasks that intend to trigger emotional responses have been developed and implemented in clinical research. It is possible, that BOLD responses elicited during these other paradigms show better repeatability indices than the ones reported here.

### Summary and conclusions

We found low repeatability of activation in the amygdala and fusiform gyrus during emotion processing, as had been previously reported. We also showed here that repeatability of the signal amplitude did not improve by accounting for physiological noise or day-to-day differences in BOLD responsiveness as assessed with a breath-holding task. This indicates that other unaccounted-for sources of noise are more influential than the ones here considered, or simply that the neural activity underlying BOLD signal is not temporally stable, questioning the utility of these measures in the study of biomarkers for mental disorders. Further research is needed to identify other potential factors that could be accounted for in order to increase the repeatability of BOLD measures in these brain areas, and therefore make them more suitable biomarkers for mental conditions.

The following are the supplementary data related to this article.

Supplementary Material **Table S1**: Correlation between BOLD responsiveness and percent signal change. This table presents correlation coefficient between the signal changes extracted from the breath-hold vs. emotion task.
**Table S2**: Influence of the correction for BOLD responsiveness on repeatability. ICCs with and without correction for BOLD responsiveness are shown for the uncorrected and physiological noise corrected dataset. Correction for BOLD responsiveness was performed by regressing out the influence of BOLD responsiveness on the emotion-task related BOLD signals, and by using the remaining signal to calculate the ICC.
**Table S3**: Variance of physiological changes explained by the task. Minimum, maximum and median R^2^ for all scans and median R^2^ for session 1 and session 2 separately are provided. Only R^2^ above .13 reach significance at *p* < .05.
**Table S4**: ROI analysis for uncorrected data when only the first half of the task is considered. For each of the ROIs, mean and standard deviation for both scanning sessions (scan 1 and scan 2), the significance of the between- session difference, and the repeatability of the value and its significance are provided.
**Fig. S1**: Group-level activation for the main effect neutral faces. Results are displayed for a significance level of Z > 2.3 and a (corrected) cluster significance threshold of *p* = 0.05. Image in radiological convention.
**Fig. S2**: Group-level activation for the main effect 50% fearful faces. Results are displayed for a significance level of Z > 2.3 and a (corrected) cluster significance threshold of *p* = 0.05. Image in radiological convention.
**Fig. S3**: Group-level activation for the main effect 100% fearful faces. Results are displayed for a significance level of Z > 2.3 and a (corrected) cluster significance threshold of *p* = 0.05. Image in radiological convention.
