# Supplementary material for: Understanding the contribution of neural and physiological signal variation to the low repeatability of emotion-induced BOLD responses
Source: Neuroimage. 2014 Feb 1;86:335–42. doi: 10.1016/j.neuroimage.2013.10.015 (PMC3898985; doi:10.1016/j.neuroimage.2013.10.015)
Supplement: Supplementary Material — Table S1: Correlation between BOLD responsiveness and percent signal change. This table presents correlation coefficient between the signal changes extracted from the breath-hold vs. emotion task. Table S2: Influence of the correction for BOLD responsiveness on repeatability. ICCs with and without correction for BOLD responsiveness are shown for the uncorrected and physiological noise corrected dataset. Correction for BOLD responsiveness was performed by regressing out the influence of BOLD responsiveness on the emotion-task related BOLD signals, and by using the remaining signal to calculate the ICC. Table S3: Variance of physiological changes explained by the task. Minimum, maximum and median R2 for all scans and median R2 for session 1 and session 2 separately are provided. Only R2 above .13 reach significance at p < .05. Table S4: ROI analysis for uncorrected data when only the first half of the task is considered. For each of the ROIs, mean and standard deviation for both scanning sessions (scan 1 and scan 2), the significance of the between- session difference, and the repeatability of the value and its significance are provided. Fig. S1: Group-level activation for the main effect neutral faces. Results are displayed for a significance level of Z > 2.3 and a (corrected) cluster significance threshold of p = 0.05. Image in radiological convention. Fig. S2: Group-level activation for the main effect 50% fearful faces. Results are displayed for a significance level of Z > 2.3 and a (corrected) cluster significance threshold of p = 0.05. Image in radiological convention. Fig. S3: Group-level activation for the main effect 100% fearful faces. Results are displayed for a significance level of Z > 2.3 and a (corrected) cluster significance threshold of p = 0.05. Image in radiological convention. [file mmc1.pdf]

Supplementary Matterial

Task-related activation - ROI analysis

Percent signal change within the amygdala did not differ between neutral, 50% fearful, and 100% fearful faces (session 1: $F[2,28] = 1.05$ *ns*; session 2: $F[2,28] = 1.49$, *ns*), and there was no interaction with hemisphere (session 1: $F[2,28] = 1.69$, ns; session 2: $F[2,28] = 0.608$, *ns*). In both sessions there was a trend for a main effect of hemisphere on activation. In session 1 the amygdala in the right hemisphere showed stronger activation ($F[1,28] = 3.36$, $p = .09$), while it was the left amygdala in session 2 ($F[1,28] = 3.80$, $p = .07$).

Within the fusiform gyrus, no significant main effects or interaction between condition and hemisphere was found in either session.

Using the dataset corrected for physiological noise did not change these results, with the exception of the trend for a main effect hemisphere disappearing in session 1, but becoming significant for session 2 ($F[1,28] = 6.89$, $p = .02$).

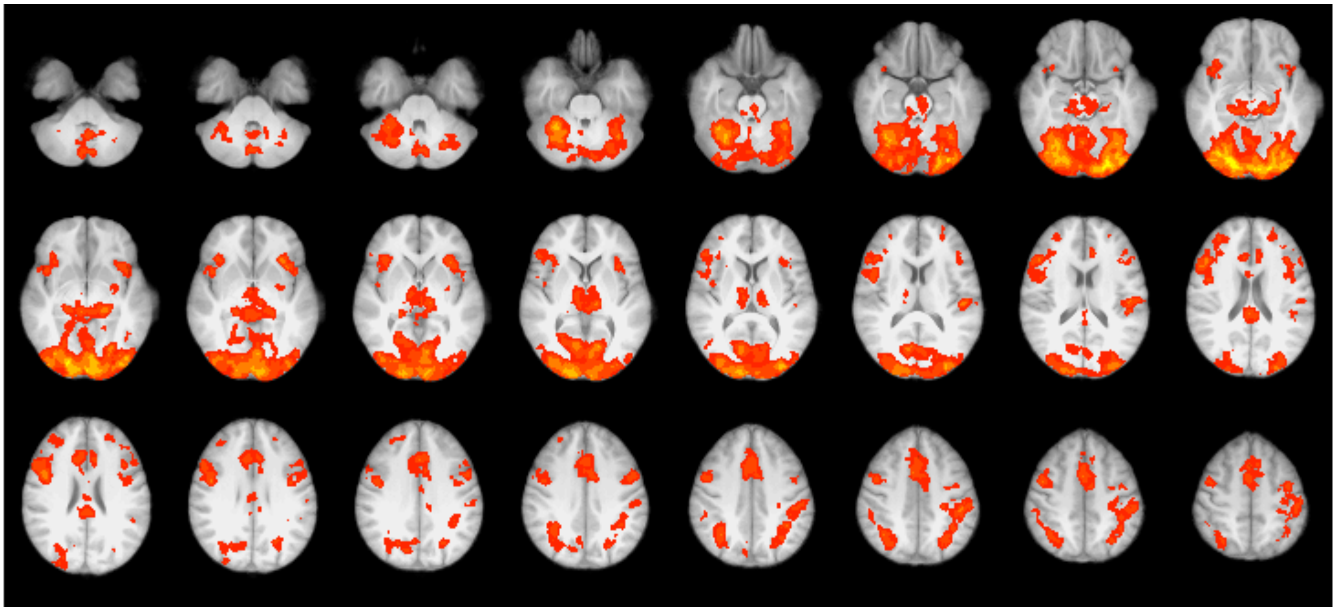

Figure S1: Group-level activation for the main effect neutral faces. Results are displayed for a significance level of Z > 2.3 and a (corrected) cluster significance threshold of P=0.05. Image in radiological convention.

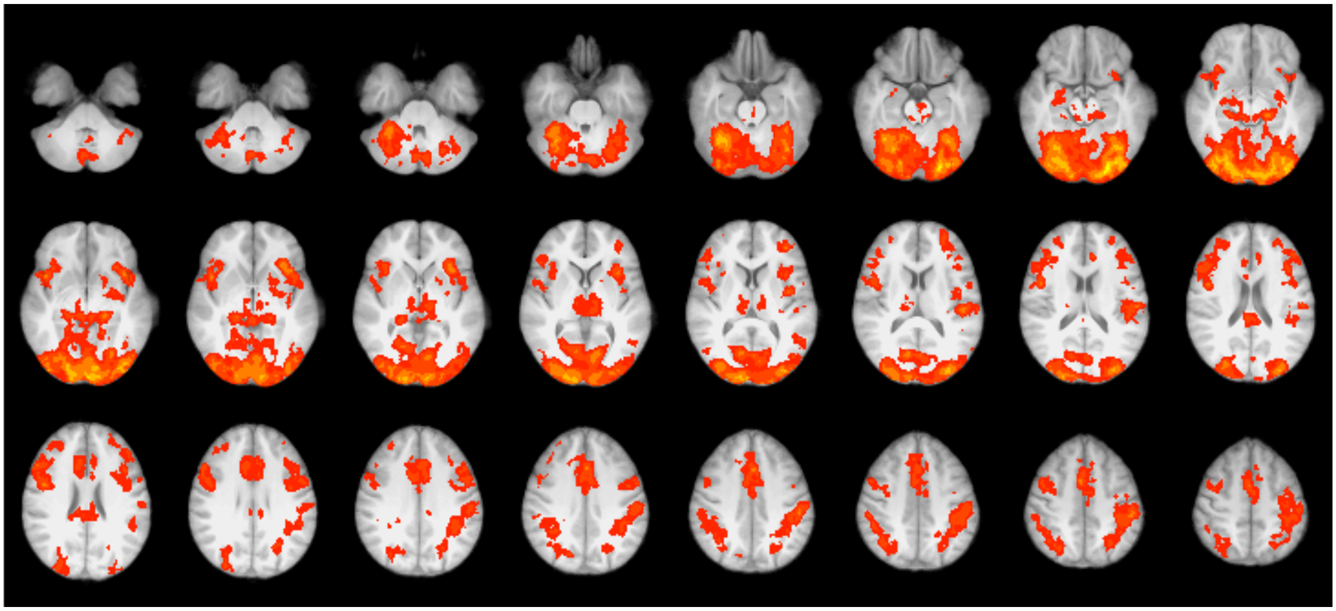

Figure S2: Group-level activation for the main effect 50% fearful faces. Results are displayed for a significance level of Z > 2.3 and a (corrected) cluster significance threshold of P=0.05. Image in radiological convention.

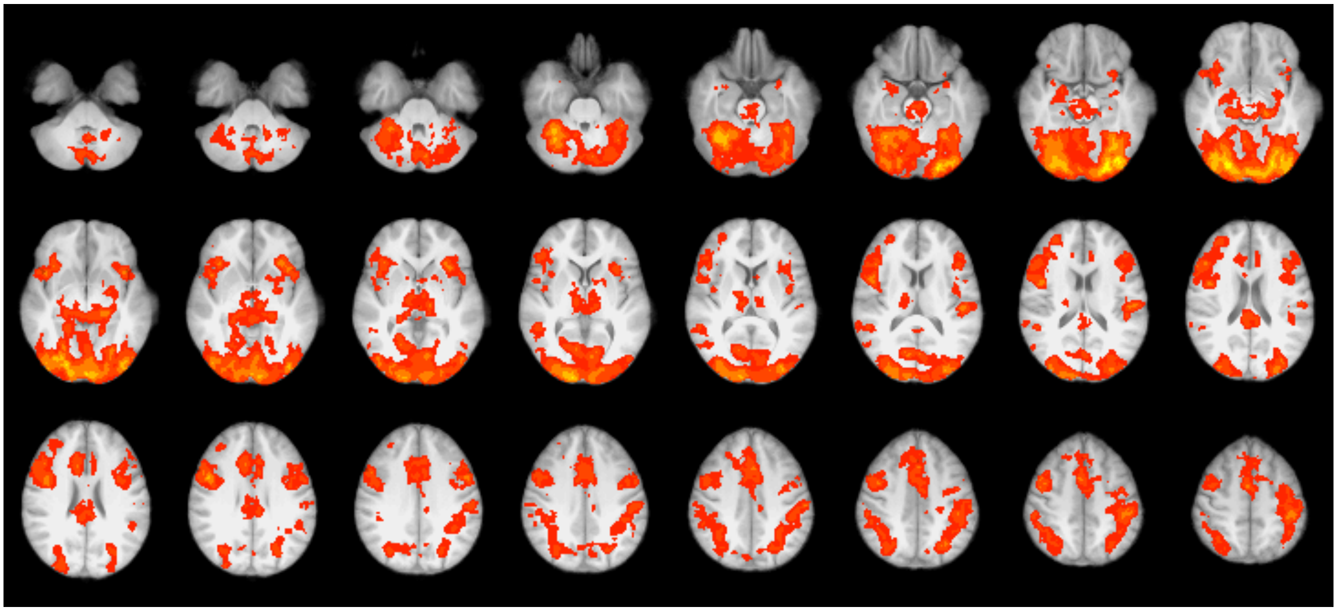

Figure S3: Group-level activation for the main effect 100% fearful faces. Results are displayed for a significance level of Z > 2.3 and a (corrected) cluster significance threshold of P=0.05. Image in radiological convention.

Table S1: Correlation between BOLD responsiveness and percent signal change. This table presents correlation coefficient between the signal changes extracted from the breath-hold vs. emotion task.

N = 14	Uncorrected data		Physiological noise corrected data	
	Scan 1	Scan 2	Scan 1	Scan 2
Left amygdala				
neutral	-.18	.42	-.19	.18
50% fear	-.17	.05	.08	.06
100 % fear	.03	.27	.19	-.03
Fear > Neutral	.16	-.27	.39	-.14
Right amygdala				
neutral	.01	.58*	.43	.46
50% fear	.49	.14	.43	.08
100 % fear	.07	.19	.34	.10
Fear > Neutral	.26	-.51	.07	-.51
Left fusiform				
neutral	-.27	.60*	-.45	.36
50% fear	-.19	.19	-.29	.14
100 % fear	-.42	.25	-.40	.23
Fear > Neutral	.05	-.43	.07	-.28
Right fusiform				
neutral	-.14	.26	-.24	.05
50% fear	-.11	-.32	-.16	-.34
100 % fear	-.21	-.18	-.27	-.14
Fear > Neutral	-.02	-.54	-.09	-.37

Table S2: Influence of the correction for BOLD responsiveness on repeatability. ICCs with and without correction for BOLD responsiveness are shown for the uncorrected and physiological noise corrected data set. Correction for BOLD responsiveness was performed by regressing out the influence of BOLD responsiveness on the emotion-task related BOLD signals, and by using the remaining signal to calculate the ICC.

N = 14	Uncorrected data		Physiological noise corrected data	
	Without BR-regression	With BR regression	Without BR regression	With BR regression
Left amygdala				
neutral	.06 (.41)	.15 (.29)	-.04	.01
50% fear	.00	-.06	-.11	-.06
100 % fear	.34 (.10)	.34 (.11)	.28 (.16)	.32 (.12)
Fear > Neutral	-.54	-.46	-.65	-.61
Right amygdala				
neutral	.00	-.12	-.06	.07 (.40)
50% fear	.39 (.08)	.37 (.09)	.19 (.25)	.28 (.16)
100 % fear	.30 (.14)	.24 (.19)	.17 (.27)	.22 (.22)
Fear > Neutral	-.52	-.56	-.44	-.34
Left fusiform				
neutral	.13 (.32)	.30 (.14)	0	.08 (.40)
50% fear	-.32	-.29	-.23	-.22
100 % fear	-.39	-.50	-.28	-.36
Fear > Neutral	-.10	-.15	-.05	-.09
Right fusiform				
neutral	.14 (.31)	.13 (.33)	.07 (.40)	.04 (.45)
50% fear	-.04	-.08	-.07	-.16
100 % fear	-.05	-.12	-.07	-.17
Fear > Neutral	-.03	-.10	.11 (.35)	.10 (.36)

Table S3: Variance of physiological changes explained by the task. Minimum, maximum and median R^2 for all scans and median R^2 for session 1 and session 2 separately are provided. Only R^2 above .13 reach significance at $P < .05$.

Condition	Min. R^2	Max. R^2	Median R^2	Median R^2 scan 1	Median R^2 scan 2
all conditions	.03	.16	.08	.06	.09
neutral	.02	.21	.12	.11	.12
50% fear	.03	.17	.09	.10	.09
100 % fear	.03	.23	.09	.08	.09
Fear > neutral	.03	.20	13	.13	.13

Table S4: ROI analysis for uncorrected data when only first half of the task is considered. For each of the ROIs, mean and standard deviation for both scanning sessions (scan 1 and scan 2), the significance of the between- session difference, the repeatability of the value and its significance are provided.

	No correction				Physiological noise correction			
Area/condition	*M*(*SD*) scan 1	*M*(*SD*) scan 2	*p*(diff)	ICC(*p*)	*M*(*SD*) scan 1	*M*(*SD*) scan 2	*p*(diff)	ICC(*p*)
Left amygdala:								
neutral	0.16 (0.45)	0.17 (0.38)	.92	.39 (.07)	0.16 (0.28)	0.16 (0.32)	.98	-.20
50% fearful	0.27 (0.13)	0.19 (0.31)	.29	.24 (.18)	0.26 (0.19)	0.20 (0.28)	.45	.16 (.28)
100% fearful	0.33 (0.39)	0.25 (0.25)	.40	.39 (.07)	0.23 (0.28)	0.20 (0.31)	.80	-.15
Fear > Neutral	0.38 (0.82)	0.16 (0.30)	.31	.16 (.28)	0.24 (0.72)	0.14 (0.50)	.69	-.20
Right amygdala:								
neutral	0.16 (0.33)	0.03 (23)	.14	.30 (.13)	0.13 (0.20)	0.10 (0.24)	.75	-.33
50% fearful	0.26 (0.19)	0.02 (0.21)	< .01	.42 (.05)	0.26 (0.18)	0.10 (0.19)	.03	.14 (.31)
100% fearful	0.31 (0.26)	0.05 (0.18)	.02	-.34	0.24 (0.25)	0.09 (0.21)	.10	.09 (.37)
Fear > Neutral	0.32 (0.55)	0.02 (0.45)	.15	-.12	0.29 (0.42)	0.01 (0.46)	.16	-.38
Left fusiform:								
neutral	0.25 (0.35)	0.19 (0.30)	.56	.20 (.23)	0.28 (0.28)	0.11 (0.32)	.20	-.28
50% fearful	0.39 (0.23)	0.13 (0.46)	.09	-.16	0.39 (0.18)	0.11 (0.33)	.02	-.24
100% fearful	0.46 (0.32)	0.18 (0.28)	.07	-.58	0.35 (0.24)	0.14 (0.21)	.07	-.59
Fear > Neutral	0.47 (0.72)	-0.02 (0.47)	.09	-.40	0.30 (0.48)	0.07 (0.44)	.28	-.41
Right fusiform:								
neutral	0.31 (0.42)	0.27 (0.18)	.73	.06 (.41)	0.32 (0.29)	0.18 (0.25)	.24	-.40
50% fearful	0.39 (0.21)	0.17 (0.36)	.10	-.30	0.42 (0.19)	0.14 (0.27)	.01	-.29
100% fearful	0.48 (0.39)	0.21 (0.24)	.08	-.51	0.40 (0.31)	0.18 (0.18)	.08	-.53
Fear > Neutral	0.38 (0.76)	-0.10 (0.44)	.10	-.43	0.29 (0.38)	0.00 (0.35)	.11	-.57
